# Identifying Systematic Force Field Errors Using a 3D-RISM Element Counting Correction

**DOI:** 10.3390/molecules28030925

**Published:** 2023-01-17

**Authors:** Lizet Casillas, Vahe M. Grigorian, Tyler Luchko

**Affiliations:** Department of Physics and Astronomy, California State University, Northridge, CA 91330, USA

**Keywords:** solvation, hydration free energy, 3D-RISM, force field, volume correction, conformational sampling, generalized Born, Lennard–Jones, implicit solvent, partial molar volume

## Abstract

Hydration free energies of small molecules are commonly used as benchmarks for solvation models. However, errors in predicting hydration free energies are partially due to the force fields used and not just the solvation model. To address this, we have used the 3D reference interaction site model (3D-RISM) of molecular solvation and existing benchmark explicit solvent calculations with a simple element count correction (ECC) to identify problems with the non-bond parameters in the general AMBER force field (GAFF). 3D-RISM was used to calculate hydration free energies of all 642 molecules in the FreeSolv database, and a partial molar volume correction (PMVC), ECC, and their combination (PMVECC) were applied to the results. The PMVECC produced a mean unsigned error of 1.01±0.04kcal/mol and root mean squared error of 1.44±0.07kcal/mol, better than the benchmark explicit solvent calculations from FreeSolv, and required less than 15 s of computing time per molecule on a single CPU core. Importantly, parameters for PMVECC showed systematic errors for molecules containing Cl, Br, I, and P. Applying ECC to the explicit solvent hydration free energies found the same systematic errors. The results strongly suggest that some small adjustments to the Lennard–Jones parameters for GAFF will lead to improved hydration free energy calculations for all solvent models.

## 1. Introduction

Hydration free energy (HFE) and, more generally, solvation free energy of small molecules are important quantities for predicting the physical properties of small molecules, such as hydrophobicity, solubility, and molecular binding. Physics-based molecular simulation is a powerful tool for such calculations, and several approaches have been developed with various levels of detail and computational demands. Ref. [1] Amongst the most common approaches is to treat the solute with an all-atom, force-field-based model, which is then coupled with a solvent model and an appropriate method to calculate the HFE When coupled with an explicit all-atom solvent model, thermodynamic integration or free energy perturbation are commonly used to calculate the HFE. This approach is computationally expensive as molecular dynamics simulations are required for each solute, each of which requires adequate conformational sampling. Alternatively, implicit solvents, such as Poisson–Boltzmann (PB) [2] or generalized Born (GB) [3], may be used. These models typically provide significant computational savings, as the detail of the solvent model is significantly reduced and, if the solute is sufficiently rigid, conformational sampling is not required, and only calculations in the aqueous phase are often all that is needed. The 3D reference interaction site model (3D-RISM) [4,5] of molecular solvation is an implicit solvent of particular interest because it uses all-atom force fields to model the solvent, but utilizes statistical mechanics via an approximated Ornstein–Zernike (OZ) equation [6] to calculate equilibrium distributions. As such, it produces complete solvation thermodynamic information in a single calculation, and avoids thermodynamic integration or free energy perturbation of rigid solutes. However, the accuracy of calculated HFEs depends on both models of the solvent and solute, both of which may have systematic errors.

It is well known that 3D-RISM overestimates HFEs for small molecules, and several post-calculation corrections have been developed. Refs. [7,8,9,10,11] These corrections have been shown to produce HFEs of similar quality to explicit solvent calculations, and have been extended to correct entropy [12] as well as other solvents [13,14,15,16,17,18,19]. While other approaches exist, such as creating new bridge functionals [11] or using hard-sphere liquids as a reference [20], most have employed corrections based on the partial molar volume (PMV). Recently, a correction using van der Waals (vdW) volumes instead of PMVs was proposed, and improved agreement with the experiment was demonstrated on a rigid subset of molecules from the FreeSolv database [10,21]. However, calculating the van der Waals volume was not based on physical principles, but was fit for each element to provide maximum agreement with the experiment. This correction has a free parameter for each element in the dataset, compared to only two free parameters used in the original PMV correction by Palmer et al. [9]. At the same time, the flexibility provided by this element-based approach may be of practical use in improving the solute force field employed when coupled with any solvation model.

In this paper, we simplify the vdW volume correction and combine it with PMV corrections in 3D-RISM, and apply it to explicit solvent simulations. First, we show that the vdW correction can be reproduced by counting the number of atoms of each element in the solute rather than calculating a volume. This requires minimal computation, is trivial to implement, and, as we will show, performs as well as methods using the vdW or partial molar volume. We then extend the correction by first combining it with the Palmer et al. [9] PMV correction and, separately, use it to improve the HFEs from the explicit solvent calculations of Mobley et al. [22,23]. The observed improvements suggest that this correction may address underlying deficiencies in the solute force field.

We begin by reviewing the theoretical basis of the PMV and vdW volume corrections in Section 2. We then provide results for the various corrections used with both 3D-RISM and explicit solvent for both rigid and flexible subsets of the FreeSolve database in Section 3. In Section 4, we discuss our results in the context of other corrections. In Section 5, we provide details of the calculations.

## 2. Theory

Details of the 3D-RISM theory have been provided elsewhere [24,25]. Here, we focus on corrections applied after a 3D-RISM calculation has been carried out and the solvation free energy has been calculated.

### 2.1. PMV Correction

The primary source of error in 3D-RISM calculations appears to be due to extreme overestimation of pressure in the model, which is typically around 1000 times too high for water at ambient density and temperature [26]. This pressure artifact is likely due to the closure approximation employed when solving 3D-RISM, as pressure consistency approaches have been shown to improve both the pressure and excess chemical potential in OZ calculations [27]. Indeed, the closures most commonly used with 3D-RISM are the Kovalenko–Hirata (KH) [28], partial series expansion of order-*n* (PSE-*n*) [29], and the Kobryn–Guasrov–Kovalenko (KGK) closures [30], which are approximations of the hypernetted chain equation (HNC) [31], known to overestimate the pressure [32]. This was first addressed by Palmer et al. [9] as a simple linear correction,

(1) $$\Delta G_{\mathrm{PMVC}}=\Delta G_{\mathrm{RISM}}+av+b,$$

where *v* is the PMV, and *a* and *b* are free parameters to be fit against the experiment. In this form, *a* can be interpreted as the overestimation of the pressure, and is negative when fit to experiment. However, *a* does not numerically correspond to the pressure calculated from compressibility or the free energy routes. It has been suggested that *a* corresponds to the contact pressure [8,33], but this approach does not work for the KH closure [12] or non-spherical molecules [13].

### 2.2. vdW Volume Correction

Robert et al. [10] proposed a pressure correction and the vdW volume (PCvdW) of each solute site with the pressure from the free energy route,

(2) $$\begin{array}{cc} \Delta G_{\mathrm{PCvdW}} & =\Delta G_{\mathrm{RISM}}+P_{\mathrm{FE}}V_{\mathrm{vdW}} \end{array}$$

(3) $$\begin{array}{cc} \; & =\Delta G_{\mathrm{RISM}}+P_{\mathrm{FE}}\sum_{i}^{N_{\mathrm{atoms}}}V_{i}, \end{array}$$

where $V_{i}$ is the vdW volume of solute atom *i*, and $P_{\mathrm{FE}}$ is the pressure from the free energy route, though any pressure can be used in practice as it is a fixed value defined by the bulk liquid. In this case, $V_{i}$ was calculated from vdW radii, by placing the solute on a grid with 0.05Å spacing and assigning voxels to particular atoms. The radii were optimized against simulation or experimentation, and were found to be close to Bondi values [34].

### 2.3. Element Count Correction

While the procedure of calculating the total vdW volume from the atomic volumes on a grid is relatively inexpensive, it can be estimated as the product of the total number, $N_{i}$, and average occupied volume, $v_{i}$, of each element, allowing a simple element count correction (ECC):

(4) $$\begin{array}{cc} \Delta G_{\mathrm{ECC}} & =\Delta G_{\mathrm{RISM}}+P_{\mathrm{FE}}\sum_{i}^{N_{\mathrm{elements}}}v_{i}N_{i}. \end{array}$$

The justification for this approach is that the number of bonds for each element is consistent, as are the bond lengths and angles, so we expect little variation in the vdW volume of an element in different chemical environments. We note that because there is overlap between the atoms, $v_{i}$ cannot be used to meaningfully calculate vdW radii. The above equation can also be expressed as

(5) $$\Delta G_{\mathrm{ECC}}=\Delta G_{\mathrm{RISM}}+\sum_{i}^{N_{\mathrm{elements}}}c_{i}N_{i},$$

where $c_{i}$ is a fit coefficient, which is simply an unspecified element-wise correction to the solvent model, solute force field, or both. It is this interpretation that we will use in this paper, and one that lends itself to being combined with PMVC, Equation (1), giving a partial molar volume with element count correction (PMVECC)

(6) $$\Delta G_{\mathrm{PMVECC}}=\Delta G_{\mathrm{RISM}}+av+b+\sum_{i}^{N_{\mathrm{elements}}}c_{i}N_{i}.$$

This correction can account for both systematic errors in 3D-RISM as well as issues with individual elements, though at the cost of using considerably more parameters than the PMVC.

## 3. Results

### 3.1. Identifying Rigid and Flexible Molecules Using Molecular Dynamics with GB Solvent

All 3D-RISM calculations in this study use a single conformer of the solute. To assess and mitigate errors due to this approach, we classified the solutes in the FreeSolv database as ‘flexible’ or ‘rigid’ for separate analysis. In this case, we are interested in flexibility as it relates to the HFE; however, it is computationally demanding to obtain correctly sampled conformers. To address this, we carried out MD simulations using GB as the solvent, which allows for extensive sampling at modest computational cost and provides HFEs, $\Delta G_{\mathrm{GB}}$, for individual conformers from the simulation. Molecules that are rigid should have low standard deviations in $\Delta G_{\mathrm{GB}}$ over the course of a simulation, and this should be reflected in a small difference between $\Delta G_{\mathrm{GB}}$ calculated from the entire simulation, $\Delta G_{\mathrm{GB},\mathrm{MD}}$, and the first frame only ($\Delta G_{\mathrm{GB},\mathrm{static}}$).

**Figure 1:**
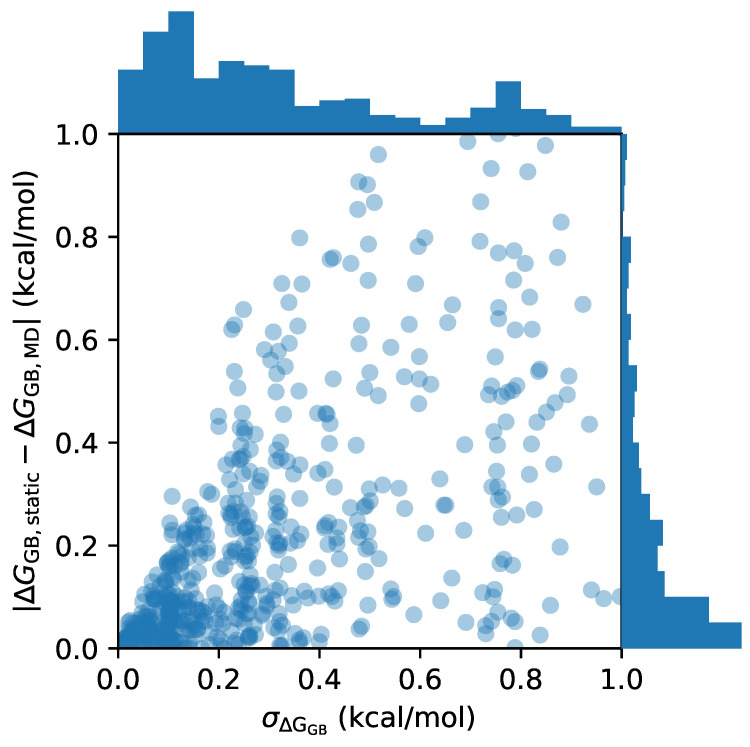
Categorizing rigid and flexible molecules from MD simulations. The standard deviation of the combined GB and surface area from MD simulations is given on the *x* axis. The difference between $E_{\mathrm{GB}}$ calculated from just the first frame (static) and over the entire MD trajectory is given on the *y* axis. Histograms for both quantities are given on their respective axes. For clarity, the full range of the data is not shown, which has maximum values of $\sigma_{\Delta G_{\mathrm{GB}}}=4.0\;\mathrm{kcal}/\mathrm{mol}$ and $\left|\Delta G_{\mathrm{GB},\mathrm{static}}-\Delta G_{\mathrm{GB},\mathrm{MD}}\right|=7.4\;\mathrm{kcal}/\mathrm{mol}$.

Figure 1 shows the distribution of $\Delta G_{\mathrm{GB}}$ standard deviations, $\sigma_{\Delta G_{\mathrm{GB}}}$, over the course of the simulations, and $\left|\Delta G_{\mathrm{GB},\mathrm{static}}-\Delta G_{\mathrm{GB},\mathrm{MD}}\right|$. Almost all molecules with a low standard deviation, $\sigma_{\Delta G_{\mathrm{GB}}}\leq 0.4\;\mathrm{kcal}/\mathrm{mol}$, show a difference in $\Delta G_{\mathrm{GB}}$ between single snapshots and MD of no more than 0.2kcal/mol. Nonetheless, we do observe that some molecules have a poor initial configuration, and $\left|\Delta G_{\mathrm{GB},\mathrm{static}}-\Delta G_{\mathrm{GB},\mathrm{MD}}\right|$ is larger than 0.2kcal/mol despite the low $\sigma_{\Delta G_{\mathrm{GB}}}$. This demonstrates, as expected, that there is little difference between using a rigid structure and a full MD simulation if the molecules display low HFE variance. As a result, we define the 313 molecules with $\sigma_{\Delta G_{\mathrm{GB}}}\leq 0.4\;\mathrm{kcal}/\mathrm{mol}$ and $\left|\Delta G_{\mathrm{GB},\mathrm{static}}-\Delta G_{\mathrm{GB},\mathrm{MD}}\right|\leq 0.2\;\mathrm{kcal}/\mathrm{mol}$ as rigid, and the remaining 328 molecules as flexible, for the rest of the study. The full list of molecules with $\Delta G_{\mathrm{GB},\mathrm{MD}}$, $\Delta G_{\mathrm{GB},\mathrm{static}}$, $\sigma_{\Delta G_{\mathrm{GB}}}$, and rigid/flexible classification is available in Spreadsheet S1.

### 3.2. Fitting PMVC, ECC, and PMVECC Parameters

After identifying rigid and flexible molecules, 3D-RISM calculations were run on single conformers of all molecules. The PMVC, ECC, and PMVECC were fit to all the data for 3D-RISM. Only ECC was fit to explicit solvent MD calculations from the FreeSolv database [22] (Table 1). The values of the fit parameters for PMVC (Table 1) are rather close to prior results [9,12]. Many of the molecules in the FreeSolv database were also used in prior fits, but this shows that the fit is not sensitive to the molecules used.

When fit without the PMVC, the ECC parameters are all negative (Table 1). We expect these parameters to be negative and correlated with the size of the elements because uncorrected 3D-RISM overestimates the HFE in a way that can be largely addressed with the PMVC. The largest corrections are for F, Cl, Br, I, and S. With the exception of S, these are all halides, and the correction scales roughly with their size; therefore, the correction is largely a pressure correction. The correction for S is large compared to oxygen, and may indicate some discrepancy in the parameters.

The PMVECC fit shows only small changes in the PMVC parameters, *a* and *b*, compared to the PMVC-only fit, while the element-specific parameters differ greatly from the ECC-only fit (Table 1). This demonstrates that the PMVC accounts for the major errors due to overestimating the pressure, while the ECC can account for discrepancies in how the force field parameters interact with the solvent. For O, F, and S, the fit parameters are positive and the negative values are close to zero, suggesting that the force field parameters are well-tuned. Elements H, N, and C all have slightly negative values, suggesting that the interaction between water and these atoms could be improved. The remaining elements (Cl, Br, I, and P) have larger coefficients, suggesting larger adjustments are required. Of these, P requires a much larger correction, indicating that this is a particular problem for the force field parameterization.

Applying ECC to the explicit solvent calculations provides a similar picture to the PMVECC fit for 3D-RISM. Clearly, no pressure correction is required for the explicit solvent calculations, and we observe that the element coefficients are strongly correlated with those for 3D-RISM/PMVECC ($R^{2}=0.91$) (Table 1). Values for O and F are again close to zero, though S now has a larger coefficient. In contrast to PMVECC, values for H and N are close to zero, and the value for C has the opposite sign. Elements Cl, Br, and I have relatively large, negative coefficients that, while smaller than those of 3D-RISM/PMVECC, suggest that both explicit solvent and 3D-RISM would benefit from the same force field parameter adjustments. We also observe that the correction for P is positive and even larger, suggesting that this element is a particular problem.

### 3.3. Quality of Fit

**Figure 2:**
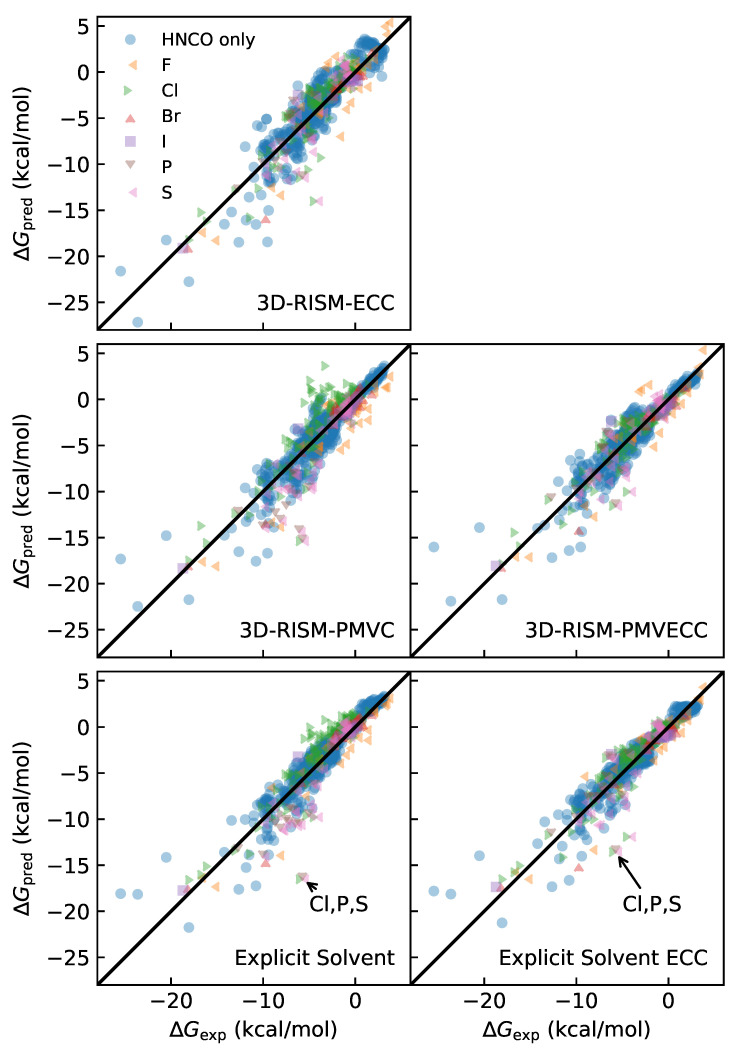
HFEs for 3D-RISM/PMVC, 3D-RISM/ECC, 3D-RISM/PMVECC, and explicit solvent using parameters from Table 1. Leave-out data were used for all plots, except for uncorrected explicit solvent calculations, which are from Refs. [22,35]. Molecules containing combinations of F, Cl, Br, P, and S atoms are plotted with multiple symbols (e.g., see labeled molecule in the bottom row). See Section 5.5 for details of the fitting procedure.

**Figure 3:**
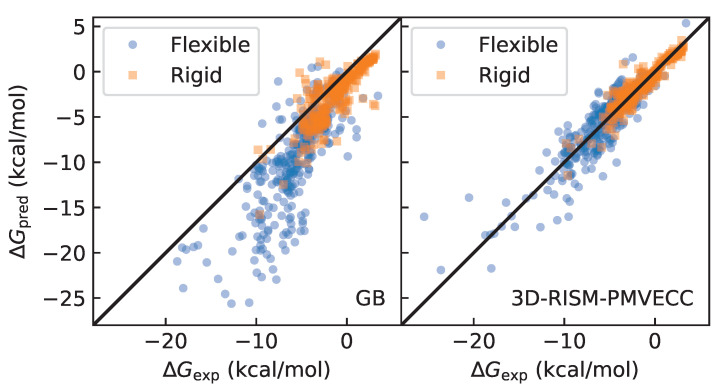
HFEs from single (original conformation) rigid and flexible datasets for GB and 3D-RISM with PMVECC.

To assess the quality of the different fits, we compared the corrected HFEs to the results from the experiment, as shown in Table 2 and Figure 2, and assess the flexible and rigid molecules separately as well as combined. Though the two datasets are nearly equal in size (313 rigid and 328 flexible), the rigid molecules are clustered in a much smaller range of HFE values (Figure 3). This is important because, for the same amount of relative error, the Pearson correlation coefficient, $R^{2}$, will be larger when computed over a smaller domain. Despite this, $R^{2}$ is larger for the rigid molecules compared to the flexible ones due to the much smaller mean unsigned errors (MUEs), root mean squared errors (RMSEs), and maximum errors for the rigid molecules. We also observe that the maximum error is much larger for the flexible molecules, even after the ECC was applied. Together, these results highlight the importance of sampling, even for small molecules, and suggest that the explicit solvent calculations may benefit from additional conformational sampling.

For all groupings of molecules, a similar picture emerges for the quality of the different corrections applied to 3D-RISM. The PMVC has lower MUE and RMSE compared to ECC, while the $R^{2}$ values are comparable. Unsurprisingly, PMVECC has the lowest errors and highest $R^{2}$ of all three corrections. In fact, PMVECC outperforms the uncorrected explicit solvent calculation, and provides similar accuracy and correlation as explicit solvent calculations with the ECC (Table 2).

A large part of the success of the PMVECC is due to correcting outliers. For example, clusters of outliers containing Cl, P, and S atoms can be observed for 3D-RISM/PMVC and uncorrected explicit solvent in Figure 2, where HFEs are overestimated for Cl-containing molecules and underestimated for P- and S-containing molecules. Accordingly, the PMVECC and explicit solvent ECC coefficients are negative for Cl and positive for P and S (Table 1), correcting the worst outliers. The ECC correction for 3D-RISM does mitigate the errors for many of these molecules, but the lack of an overall pressure correction limits its effectiveness. We also note that PMVECC effectively deals with molecules that contain multiple Cl, Br, I, P, and S atoms. For example, of the 14 P-containing molecules, 10 contain S, 5 contain Cl, and 3 contain both S and Cl. Similarly, the largest cluster of Cl outliers contain five or more Cl atoms per molecule. To illustrate the magnitude of the correction, the Cl coefficient in the PMVECC contributes −5.94kcal/mol to the HFE for a molecule with five Cl atoms.

## 4. Discussion

### 4.1. Dealing with Conformational Sampling

Insufficient conformational sampling is a common source of error when MD simulations are used [36,37], yet it is common to use single conformers of small ligands when testing or parameterizing implicit solvent models, especially for 3D-RISM (e.g., [7,9,12,14,15,16,17,18,19]). Care is required because a single-conformer approach neglects the solute adopting different conformations in a solvent than in a vacuum, conformers that are not dominant in either state, and molecules that have multiple conformers. Furthermore, the structure provided by FreeSolv or any software package may or may not be in the dominant conformation for either state. While, improving sampling for 3D-RISM is an important topic, it is beyond the scope of this study. Therefore, we wish to differentiate between rigid and flexible molecules to better assess parameter fitting and the quality of the corrections.

Solute conformational sampling, of course, can be accounted for using free energy perturbation. For example, Mobley et al. [36] connected simulations in a vacuum and GB solvents using the Bennett acceptance ratio [38]. We have previously employed a similar approach with 3D-RISM [13]; however, despite work on using 3D-RISM with MD [39,40,41,42,43,44], this approach is still computationally demanding. An alternative is to sample in a state that will overlap the conformational space in 3D-RISM and exponential averaging [45] to calculate the free energy difference. GB could be used for such an approach, but the overlap between GB and 3D-RISM conformational space is unknown and possibly insufficient to obtain accurate results.

Rather than addressing conformational sampling directly, we follow the approach of Robert et al. [10] by partitioning the dataset into flexible and rigid groups. However, our approach differs in how we categorize molecules as rigid or flexible. Robert el al. [10] compared the SFEs calculated by rigid Monte Carlo for an explicit solvent to the value computed with MD in the FreeSolv database. Those that differed by <0.1 kcal/mol were considered rigid. However, their approach required HFEs to be calculated for an explicit solvent for both completely fixed conformation and flexible solutes—an expensive calculation we would preferably avoid. Furthermore, if the right conformer is selected, the HFE of the rigid molecule could be within the threshold when compared to the flexible solute HFE, even if the molecule is rather flexible. In contrast, our approach used MD with a GB solvent, and defined molecules to be rigid if $\sigma_{\Delta G_{\mathrm{GB}}}\leq 0.4\;\mathrm{kcal}/\mathrm{mol}$ and the difference between the first frame and the HFE for the trajectory calculated by the Bennett acceptance ratio is <0.2 kcal/mol. The first criterion is a measure of how flexibility directly contributes to the HFE by utilizing the excellent computational and sampling efficiency of GB [46], while the second criterion removes molecules with poor initial conformers. In all, we define 313 molecules as rigid, compared to 288 and 520 molecules defined as rigid by Robert et al. [10] and Luukkonen et al. [21], respectively. A full list of molecules with assigned categories is available in Spreadsheet S1. The categorization of molecules as rigid or flexible was only used to assess the quality of the corrections, and not to fit the parameters, as the small range of HFEs for rigid from experiment could bias the results. Furthermore, the error due to lack of conformational sampling should be randomly distributed, so there should be no systematic effect on fitting the corrections.

From our analysis, we can see that there are larger errors for flexible molecules than for rigid ones, both for 3D-RISM and explicit solvent calculations. Two potential explanations could account for this. A constant relative error for all HFEs would lead to an increased absolute error for molecules with large magnitude HFEs. Flexible molecules have a wider distribution of HFE values, and would be expected to have larger absolute errors on average. Alternatively, flexible molecule conformations may be undersampled, even in explicit solvent MD. We can test this by considering the relative error,

$$\epsilon =\left|\frac{\Delta G_{\mathrm{pred}}-\Delta G_{\exp}}{\Delta G_{\exp}}\right|,$$

for rigid and flexible molecules. To ensure the relative error is well-behaved, we avoid dividing by a small number by only considering molecules for which $\Delta G_{\exp}<-5\;\mathrm{kcal}/\mathrm{mol}$. We find that the relative error for flexible molecules in explicit solvent is slightly lower than that for rigid molecules (22% for rigid and 20% for flexible), but in 3D-RISM/PMVECC, flexible molecules have a larger relative error (16% for rigid and 19% for flexible). Thus, the explicit solvent MD calculations are likely based on adequate sampling, whereas 3D-RISM calculations are not. Improving solute conformational sampling in 3D-RISM calculations is an important next step for increasing accuracy, even for small molecules.

### 4.2. Accuracy and Computational Efficiency of 3D-RISM/PMVECC

Of the several PMV-based HFE corrections that have been previously developed [7,8,9], we found that the original correction proposed by Palmer et al. [9] performed the best, until now [12]. By itself, we found that the ECC is of similar accuracy to PMVC, despite including many more parameters. Overall, the correlation is improved and systematic errors for specific elements are removed, though there is a slight increase in the error. Levesque and co-workers report similar statistics using their original method, which employed calculating the vdW volume instead of counting atoms [10,21]. PMVECC shows a significant improvement in both the error and the correlation to the experiment. In fact, it statistically outperforms the uncorrected explicit solvent. With recent improvements to our 3D-RISM implementation [47], the average calculation time per molecule on a single CPU core was 14.7 s and the maximum time was 38.1 s.

Though simple to implement, PMVECC does require sufficient experimental data to fit 12 parameters, potentially limiting its practical use. In addition, all or nearly all of the atoms in the FreeSolv database are solvent-accessible, and it is likely that the PMVECC correction is not transferable to molecules with many buried atoms. Future improvements in calculating HFEs will likely come from new closure approximations instead [11,27,48].

### 4.3. Force Field Parameters

A potential immediate use of the PMVECC or ECC is to correct solute force field parameters. While errors have been correlated with various solute properties, such as functional groups [21,22,49], in the absence of conformational sampling, the only force field parameters that matter are the partial charges and the Lennard–Jones parameters. Different methods of assigning partial charges may yield better results, but alternate approaches have not yet improved on AM1-BCC for GAFF [50]. A more promising approach, in our opinion, is to optimize Lennard–Jones parameters, which play a significant role in controlling the distance between the charge sites on the solute and solvent. With this in mind, Table 1 provides a clear recommendation that Lennard–Jones radii should be increased for P and S and decreased for Cl, Br, and I in the GAFF [51] used in the FreeSolv database. Generally, 3D-RISM would benefit from greater changes than for the explicit solvent, but the trend is the same in both cases.

## 5. Materials and Methods

### 5.1. Structure Preparation

For all calculations, we used the AMBER parameter and coordinate files provided with the FreeSolv v0.52 dataset [22,35]. The FreeSolv authors used GAFF for the solute force field [51] with AM1-BCC charges [52,53].

### 5.2. GB HFE

From the initial coordinates provided with the FreeSolv dataset, we ran 100 ns of MD simulation using both GB with surface area implicit solvent (igb = 2, gbsa = 1) and vacuum (igb = 6) [3,54,55] environments in the sander MD engine of AmberTools 2017 [56]. For all simulations, a 1 fs time step was used, temperature was held at 298.15K using a Langevin thermostat with $\gamma =5\;ps^{-1}$, and conformations were saved every 10,000 steps. The resulting trajectories were then post-processed in sander (imin = 5) using the GB with surface area implicit solvent (igb = 2, gbsa = 1) and in a vacuum to obtain the potential energy of each conformation in aqueous and gas phases. HFEs were then calculated from these potential energies using pyMBAR 3.1.1 [57,58].

### 5.3. 1D-RISM

Bulk properties of water at 298.15K and a concentration of 55.4M were calculated with rism1d in AmberTools 2021 [44,59]. The coincident extended simple point charge model (cSPC/E) was used to model water [44,60]. The dielectrically consistent RISM (DRISM) equations [61,62] were solved with a dielectric constant of 78.497 to a residual tolerance of $10^{-12}$ on a 16,384-point grid, with a grid spacing of 0.025Å. Convergence was accelerated with the modified inversion of iterative subspace (MDIIS) method [63].

### 5.4. 3D-RISM Calculations

rism3d.snglpnt of AmberTools 2021 [44,59] was used to calculate the HFE and PMV using the AMBER parameter and coordinate files provided with the FreeSolv dataset for each solute and bulk water properties from rism1d. The 3D-RISM equations were solved to a residual tolerance of $10^{-4}$ on a grid with spacing of 0.3Å, accelerated by MDIIS. Lennard–Jones cutoffs with a relative tolerance of $10^{-4}$ were used to determine the size of the grid and analytic corrections were applied [47]. Reciprocal space long-range asymptotics were calculated with a relative tolerance of $10^{-5}$.

### 5.5. Parameter Fitting

Parameters for the PMVC, ECC, and PMVECC were fit with leave-one-out cross-validation as follows. HFEs calculated by 3D-RISM and explicit solvent molecular dynamics [22] for the 642 molecules in the FreeSolv database were collected in a Pandas dataframe [64,65], and ordinary least squares was applied to produce 642 fits of the parameters to the data using the statsmodels package [64]. One molecule was left out for each fit in turn. Values in Table 1 are the mean of all fits and the uncertainties are the standard errors of the mean. Statistics in Table 2 were computed from the leave-out data from generating the parameters in Table 1, and uncertainties are the standard errors of the mean for 1000 rounds of bootstrap analysis. Coefficients in Table 1 and all of the leave-out data presented in Table 2 and Figure 2 can be reproduced with Script S1 in the Supplementary Materials. The corrected leave-out HFEs can also be found in Spreadsheet S1.

## Figures and Tables

**Table 1:** Fit parameters for PMVC, ECC, and PMVECC, averaged over all leave-one-out fits. Uncertainties in the last digit are given in parentheses, and represent the standard deviation over all leave-one-out fits. Uncertainty for the *a* coefficient for PMVC is $8\times 10^{-5}\;\mathrm{kcal}/\mathrm{mol}/{Å}^{3}$. Coefficient *a* is in kcal/mol/Å${}^{3}$. All other values are in kcal/mol. See Section 5.5 for details of the fitting procedure.

	PMVC	ECC	PMVECC	Explicit Solvent ECC
*a*	−0.15		−0.130(1)	
*b*	−0.04(1)		0.00(1)	
H		−1.199(1)	−0.225(5)	−0.098(1)
N		−1.573(6)	−0.392(7)	0.091(5)
C		−1.667(1)	−0.148(8)	0.114(1)
O		−1.277(3)	0.069(9)	0.088(3)
F		−2.082(4)	−0.05(1)	0.076(2)
Cl		−4.695(4)	−1.19(2)	−0.456(2)
Br		−5.544(7)	−1.06(2)	−0.412(6)
I		−6.27(1)	−0.79(3)	−0.25(1)
P		−1.03(3)	2.04(3)	2.93(3)
S		−3.18(1)	0.09(2)	0.32(1)

**Table 2:** Hydration free energies calculated with 3D-RISM and an explicit solvent [22] with PMVC, ECC, and PMVECC corrections using parameters from Table 1. Leave-out data were used to calculate statistics, except for uncorrected explicit solvent calculations, which used data from Ref. [22] with the same bootstrap procedure. All values are given in kcal/mol. Uncertainties in the last digit are given in parentheses and represent the standard error of the mean. See Section 5.5 for details of the fitting procedure.

		Slope	MUE	MSE	RMSE	$R^{2}$	Max Error
Rigid							
	3D-RISM/PMVC	0.93(4)	0.86(6)	−0.29(7)	1.3(1)	0.75(4)	6.6
	3D-RISM/ECC	0.92(4)	1.02(6)	−0.51(8)	1.37(8)	0.76(3)	5.9
	3D-RISM/PMVECC	0.92(2)	0.61(3)	0.05(5)	0.83(6)	0.89(2)	4.4
	Explicit solvent	0.96(3)	0.85(4)	−0.59(6)	1.11(6)	0.86(2)	4.6
	Explicit solvent, ECC	0.91(2)	0.66(3)	−0.14(5)	0.86(4)	0.88(1)	3.1
Flexible							
	3D-RISM/PMVC	0.98(4)	1.53(8)	0.2(1)	2.1(1)	0.75(3)	9.6
	3D-RISM/ECC	1.07(4)	1.56(7)	0.0(1)	2.1(1)	0.78(3)	9.8
	3D-RISM/PMVECC	0.95(5)	1.35(6)	−0.04(9)	1.8(1)	0.79(3)	9.4
	Explicit solvent	0.97(4)	1.34(7)	−0.09(0)	1.8(1)	0.79(3)	10.8
	Explicit solvent, ECC	0.91(4)	1.17(6)	−0.13(9)	1.7(1)	0.81(3)	7.8
Total							
	3D-RISM/PMVC	1.01(3)	1.22(5)	0.00(7)	1.77(9)	0.83(2)	9.6
	3D-RISM/ECC	1.06(2)	1.32(5)	−0.21(7)	1.80(8)	0.84(2)	9.8
	3D-RISM/PMVECC	0.96(3)	1.01(4)	0.00(6)	1.44(7)	0.87(1)	9.4
	Explicit solvent	1.02(3)	1.11(4)	−0.32(6)	1.53(8)	0.87(1)	10.8
	Explicit solvent, ECC	0.94(2)	0.94(4)	−0.13(5)	1.35(8)	0.88(1)	7.8

## Data Availability

The data presented in this study are available in the Supplementary Material.

## Supplementary Materials

The following are available online at https://www.mdpi.com/article/10.3390/molecules28030925/s1. Spreadsheet S1: HFEs from all methods (kcal/mol), element counts, and PMVs (Å${}^{3}$) for each molecule in the FreeSolv database. Script S1: Python script to calculate correction parameters in Table 1 and corrected HFEs presented in Table 2 and Figure 2.
